# Renin–angiotensin–aldosterone system inhibitors and survival in patients with hypertension treated with immune checkpoint inhibitors

**DOI:** 10.1016/j.ejca.2021.12.024

**Published:** 2022-01-19

**Authors:** Zsofia D. Drobni, Olivier Michielin, Thiago Quinaglia, Daniel A. Zlotoff, Leyre Zubiri, Hannah K. Gilman, Sama Supraja, Bela Merkely, Veronika Muller, Ryan J. Sullivan, Kerry L. Reynolds, Michael J. Pittet, Rakesh K. Jain, Tomas G. Neilan

**Affiliations:** a Heart and Vascular Center, Semmelweis University, Budapest, Hungary; b Cardiovascular Imaging Research Center (CIRC), Department of Radiology and Division of Cardiology, Massachusetts General Hospital, Harvard Medical School, Boston, MA, USA; c Oncology Department, Precision Oncology Center, Lausanne, Switzerland; d Oncology Department, Lausanne University Hospital, Lausanne, Switzerland; e Cardio-Oncology Program, Division of Cardiology, Department of Medicine, Massachusetts General Hospital, Harvard Medical School, Boston, MA, USA; f Division of Oncology and Hematology, Department of Medicine, Massachusetts General Hospital, Harvard Medical School, Boston, MA, USA; g Department of Pulmonology, Semmelweis University, Budapest, Hungary; h Department of Pathology and Immunology, University of Geneva, Geneva, Switzerland; i Edwin L. Steele Laboratories, Department of Radiation Oncology, Massachusetts General Hospital and Harvard Medical School, Boston, MA, USA

**Keywords:** Immune checkpoint inhibitors, Immune therapy, Renin–angiotensin–aldosterone system blocker, ACEI, ARB

## Abstract

**Background:**

Preclinical studies indicate that the concurrent use of inhibitors of the renin–angiotensin–aldosterone system (RAAS) may improve outcomes in broad groups of patients with cancer. There are limited data on the association between the use of RAAS inhibitors and outcomes among patients treated with immune checkpoint inhibitors (ICIs).

**Methods:**

We performed a retrospective study of all patients treated with an ICI in a single academic network. Of 10,903 patients, 5910 were on any anti-hypertensive medication. Of those on anti-hypertensive therapy, 3426 were prescribed a RAAS inhibitor during ICI treatment, and 2484 were prescribed other anti-hypertensive medications. The primary outcome was overall survival in the entire cohort and in sub-groups by cancer types.

**Results:**

Thoracic cancer (34%) and melanoma (16%) were the most common types of cancer. Those prescribed a RAAS inhibitor were older, more frequently male, and had more cardiovascular risk factors. In a Cox proportional hazard model, the concurrent use of RAAS inhibitors was associated with better overall survival (hazard ratio (HR):0.92, [95% Confidence Interval (CI):0.85–0.99], *P* = .032). Patients with gastrointestinal (HR:0.82, [95% CI: 0.67–1.01], *P* = .057) and genitourinary cancer (HR:0.81, [95% CI:0.64–1.01], *P* = .067) had a non-statistically significant better overall survival.

**Conclusions:**

In this large retrospective study, patients with hypertension who were concomitantly taking a RAAS inhibitor during ICI therapy had better overall survival. This benefit was primarily noted among patients with gastrointestinal and genitourinary cancers. Prospective randomized trials are warranted to further evaluate and specify the benefit of RAAS inhibitors in patients with cancer who receive ICI therapy.

## 1. Introduction

The use of immune checkpoint inhibitors (ICIs) has resulted in durable tumour responses among patients with a variety of cancers. In recent years, the value of an ICI has expanded from late-stage to the adjuvant and neoadjuvant settings [1,2]. It is estimated that approximately 36% of patients with cancer within the United States alone may be eligible for ICI [3]. However, approximately 20% of the patients benefit from ICIs [3]. Hence, novel strategies to improve the response to ICI therapy are needed. Preclinical studies suggest that the renin–angiotensin–aldosterone system (RAAS) plays an important role in tumour biology [4–6]. In the tumour microenvironment, RAAS may enhance immunosuppression via multiple mechanisms [4,5,7,8]. Specifically, RAAS inhibitors may reduce solid stress in tumours resulting in increased vascular perfusion, leading to improved drug and oxygen delivery to tumours through this physical mechanism, thereby potentiating standard cytotoxic chemotherapy as well as ICI therapy [4,7,9]. These basic findings, as well as the benefit of adding RAAS inhibitors in a prospective phase II trial on in locally advanced pancreatic cancer [10], and multiple retrospective observational clinical studies [5], have led to prospective randomized trials testing whether inhibition of the RAAS can improve outcomes among patients treated with traditional cytotoxic chemotherapy and radiation therapy as well as ICI (NCT03563248) [5,11].

The efficacy of ICIs relies on the successful trafficking of tumour-targeted T-lymphocytes from the secondary lymphoid organs, through the bloodstream, and into the tumour tissue and activating them once they accrue in the tumour microenvironment [12–14]. Resistance to ICI therapy is often associated with a low density of T-lymphocytes residing within the tumour tissue and is highly dependent on vascular perfusion [13]. Thus, there is scientific plausibility to support the potential for a beneficial effect of RAAS inhibitors in patients on an ICI. However, there are limited clinical data on the effect of RAAS inhibitors on outcomes among patients treated with an ICI [15,16]. Given the potentially significant impact on public health of these low-cost and relatively safe interventions, we performed a retrospective cohort study to evaluate the effect of the concurrent use of ICI and RAAS inhibitors in a large cohort of patients with cancer. We also tested the effect of different types of RAAS inhibitors, angiotensin-converting enzyme inhibitors (ACEIs) and angiotensin receptor blockers (ARBs), on overall survival, and tested the effect of RAAS inhibitors on immune-related adverse events (irAEs).

## 2. Methods

The data, analytic methods and study materials will be made available from the corresponding author on reasonable request after institutional approval and following institutional process.

### 2.1. Study design, setting and population

To assess the effect of RAAS inhibitors on ICI therapy, we performed a retrospective study. All individuals treated with an ICI through the end of August 2020 at a single academic network (Mass General Brigham, Boston, MA, USA) were included (n = 10,903). The use of an ICI was derived from a pharmacy database. The start date was defined as the first date when an ICI was administered. Other covariates of interest were derived from the Research Patient Data Registry (RPDR). These included patient demographics, medications, standard cardiovascular risk factors, vital parameters and laboratory results. Data relevant to cancer included the type of cancer, prior potentially cardiotoxic cancer therapies and detailed description of ICI treatments. The date of death was obtained from RPDR.

**Fig. 1.**
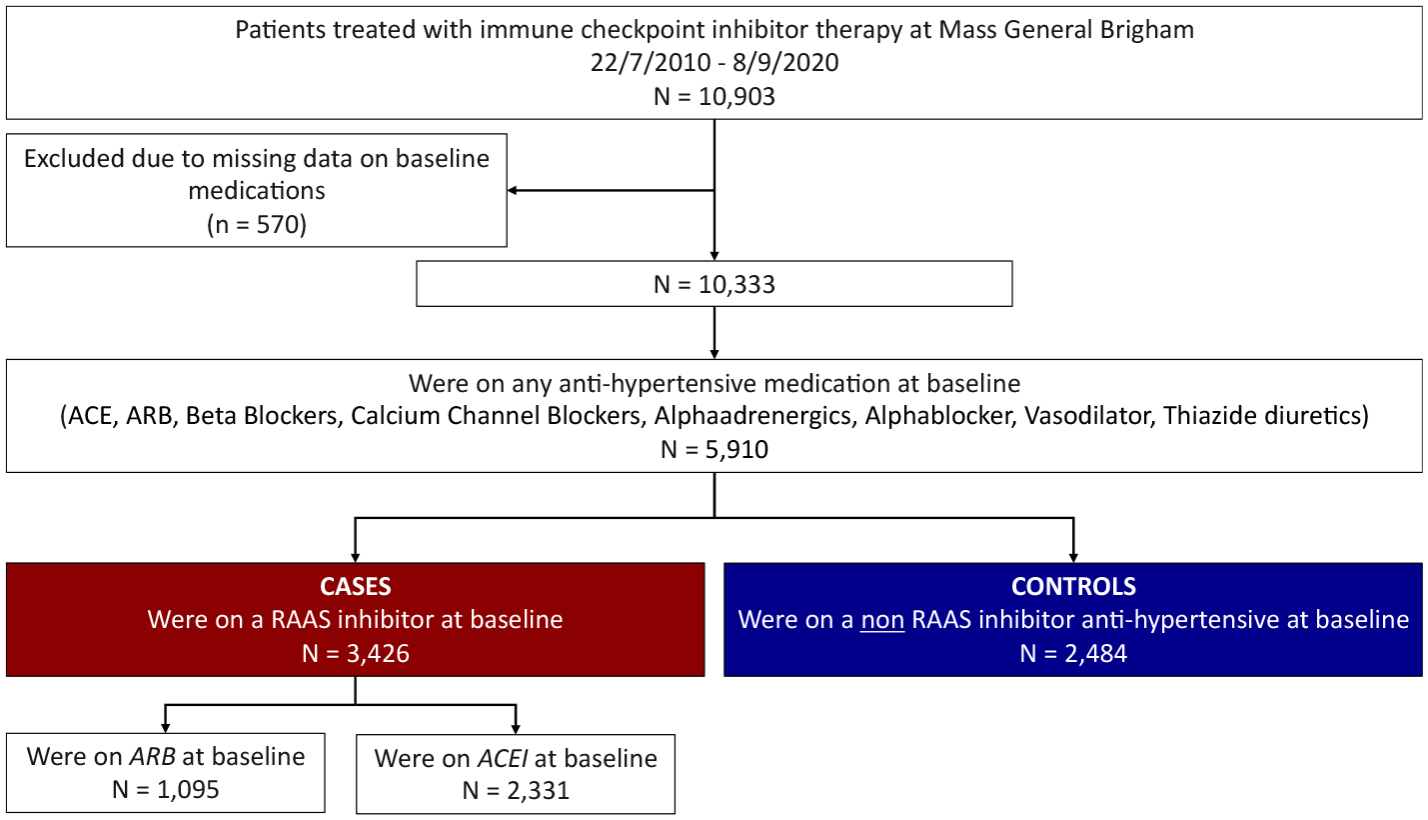
Flow chart. ACEI: Angiotensin-converting-enzyme inhibitor, ARB: Angiotensin II receptor blocker.

From this cohort of 10,903 patients, patients with missing data on baseline medications (n = 570) were excluded, which resulted in a cohort of 10,333 patients. Of these, 5910 were on an anti-hypertensive medication (Fig. 1).

Those on an inhibitor of the RAAS system were defined as patients on any ACEI or ARB at the start of ICI treatment (n = 3426), and these patients were compared with those on any other anti-hypertensive medication at the time of the ICI start (n = 2484). The study was approved by the Partners Human Research Committee and no informed consent was required. The authors vouch for the completeness and accuracy of the data and all analyses.

### 2.2. Procedures and outcomes

Covariates of interest obtained included patient demographics, medications and standard cardiovascular risk factors (e.g. diabetes mellitus, hypertension, smoking). Data specific to RAAS inhibitors also included the type and dose of RAAS inhibitors. Data relevant to cancer included the cancer type and prior potentially cardiotoxic cancer therapies (platinum-based therapy, 5-fluorouracil and anthracyclines). The use of vascular endothelial growth factor (VEGF) inhibitors was also collected. Data specific to ICI therapy also included the number of ICI cycles, the type of ICI therapy and the use of combined immune checkpoint therapy. The occurrence of irAEs was identified using ICD-10 codes.

The primary outcome was overall survival. When the date of death was not available, patients were censored at the last date of follow-up alive. Among the control patients, those who started RAAS inhibitor therapy during the study period were censored when they started RAAS inhibitor therapy.

### 2.3. Statistical analysis

Continuous variables are presented as mean (standard deviation) or median (25%–75% percentile). Categorical variables are presented as counts and percentages. Univariate Cox proportional hazard regression analysis was performed to calculate hazard ratios (HRs) with 95% confidence intervals (CI) for the full cohort and for each cancer type. Multivariable Cox proportional hazard regression analysis was performed including known risk factors associated with death (age, gender, body mass index, congestive heart failure, diabetes, renal disease, liver disease and smoking). The proportional hazard assumption was tested with the use of log–log plots and examination of Schoenfeld residuals. We performed subgroup analyses of HRs by cancer types. As a sensitivity analysis, a 1:1 propensity score matching was performed in a subset of patients using the MatchIt package, with a generalized linear model and calliper of 0.2 without replacement. The following variables were used to create propensity scores: age, gender, body mass index, congestive heart failure, diabetes, renal disease, liver disease, history of smoking and cancer type. Patients with missing data for the propensity score were excluded from the matching. Standardized mean differences were used to examine the balance of covariate distribution between the groups. For patients with gastrointestinal and genitourinary cancer, a cancer type-based propensity score matching was performed using the same variables and settings. As a second sensitivity analysis, we excluded patients with known dead status but without exact date of death (n = 559). The rates of irAEs were compared between groups. In a subset of patients where detailed data was available on the type and dose of RAAS inhibitors, univariable Cox proportional hazard models were performed to evaluate the effect of these on overall survival. The impact of VEGF inhibitor therapy in combination with RAAS inhibitors was also evaluated. All statistical tests were two-tailed, and *P* values of less than .05 were considered to indicate statistical significance. Analyses were performed with R studio software (version 1.4.1106).

## 3. Results

### 3.1. Patient demographics, comorbidities and cancer data

Baseline demographics and clinical characteristics of patients taking any anti-hypertensive medications and ICI therapy are summarized in Table 1. Thoracic cancer (34% [1950/5714]) and melanoma (16% [913/5714]) were the most common types of cancer. Programmed cell death protein 1 (PD-1) inhibitor therapy was the most commonly prescribed ICI (76% [4483/5910]), followed by programmed death-ligand 1 (PD-L1) (13% [768/5910]), and cytotoxic T-lymphocyte-associated protein 4 (CTLA-4) (3.7% [216/5910]). Overall, 7.5% of patients were on a combination of ICIs. Patients were treated for a median of 5 ICI cycles (IQR: 3–11). Those on inhibitors of the RAAS system were older, predominately male, had higher number of cardiovascular risk factors, and were more likely to have known cardiovascular diseases. Baseline laboratory parameters are reported in Table 2. Among those on a RAAS inhibitor, 68% (2331/3426) were on an ACE inhibitor, with lisinopril (89% [2077/2331]) being the most common. In total, 32% (1095/3426) were prescribed an ARB, and most were prescribed losartan (68% [748/1095]).

Detailed data regarding the daily dose of RAAS inhibitors were available in 76% of patients (2607/3426). High-dose lisinopril was defined as daily dose above the median dose, >20 mg per day. Among those on lisinopril, 45.7% were taking a high dose of lisinopril (710/1552), and 54.3% were prescribed a lower dose (842/1552). High-dose losartan was defined as daily dose above the median dose, >50 mg per day. Among those on losartan (611/2607), 35.4% were prescribed a high dose (216/611), and 64.6% were prescribed a lower dose (395/611).

Baseline characteristics for the sensitivity analysis groups are reported in the Supplementary Table 1.

### 3.2. Association between RAAS inhibitors and overall survival

**Fig. 2.**
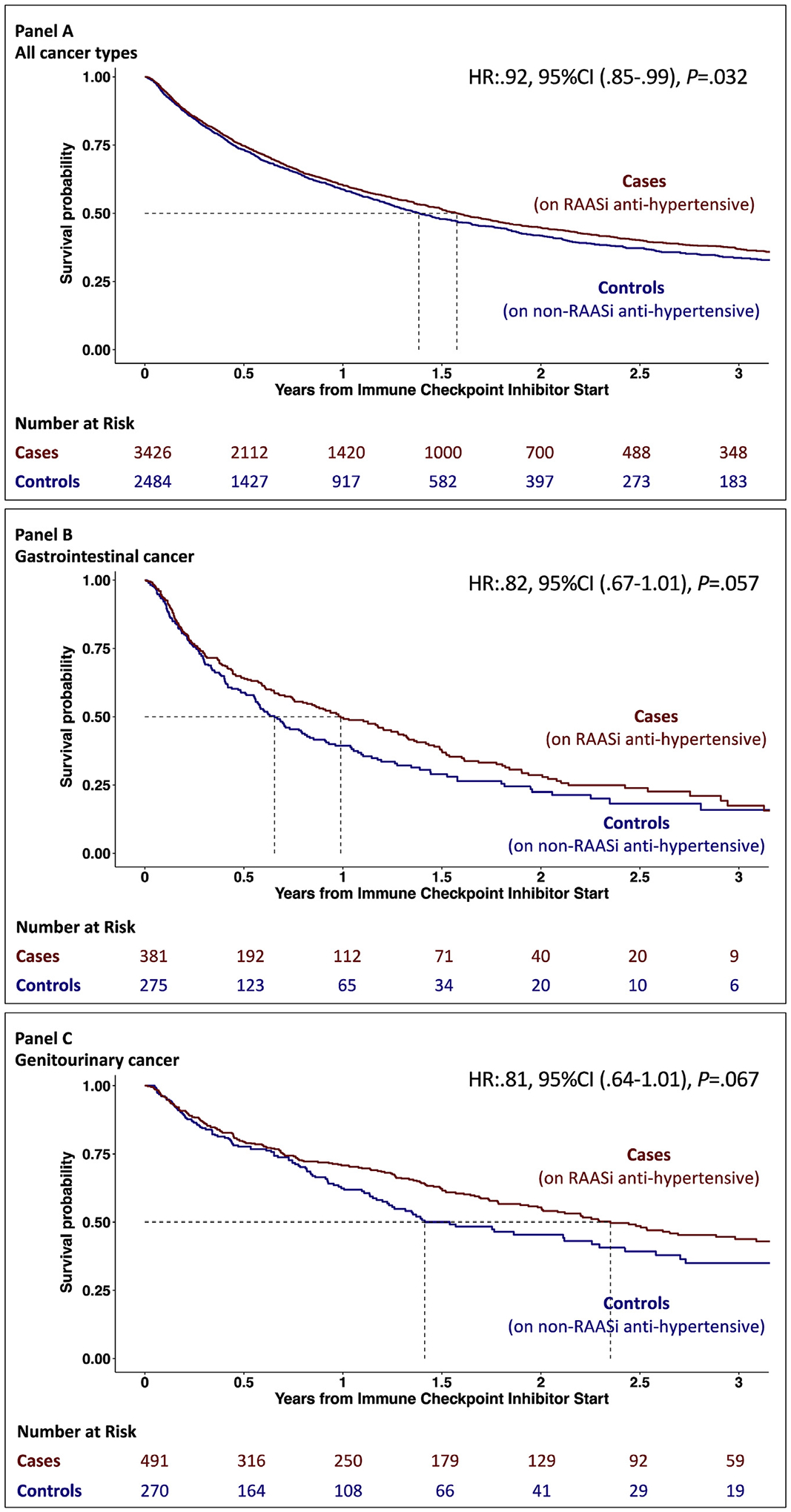
Kaplan–Meier curves of the survival probability over time after starting immune checkpoint inhibitor therapy. Panel A shows the cumulative hazard for overall survival. Cases (patients treated with RAAS inhibitors for hypertension) are marked with red, and controls (patients treated with non–RAAS inhibitors for hypertension) are marked with blue. Panel B shows the subgroup of patients with gastrointestinal cancer, and Panel C shows patients with genitourinary cancer.

Univariate analysis showed that the concurrent use of RAAS inhibitors was associated with better overall survival in the full cohort (univariate HR:.92, [95%CI:0.85–0.99], *P* = .032) (Fig. 2A).

A trend was noted toward better overall survival among patients with gastrointestinal (univariate HR:.82, [95%CI: 0.67–1.01], *P* = .057) (Fig. 2B) and genitourinary cancer (univariate HR:.81, [95%CI: 0.64–1.01], *P* = .067) (Fig. 2C). Additional analysis and Kaplan–Meier curves regarding other cancer types are shown in Supplementary Figure 1. There was no difference in survival among those on RAAS inhibitors in patients with thoracic malignancies (univariate HR:1.00, [95%CI: 0.88–1.14], *P* =.98, Supplement) or melanoma (univariate HR:1.02, [95%CI: 0.81–1.27], *P* = .89, Supplement). When we excluded patients with gastrointestinal and genitourinary cancer from the full cohort, no benefit was associated with RAAS inhibitors (univariate HR:.96, [95%CI: 0.88–1.05], *P* = .37).

In a multivariable model (adjusted for age, gender, body mass index, congestive heart failure, diabetes, renal disease, liver disease and smoking), the use of a RAAS inhibitor was associated with a better overall survival among patients who received ICI therapy (multivariable HR:.90, [95%CI: 0.84–0.98], *P* = .013). Adjusting for the same covariates, our results reached statistical significance for gastrointestinal cancer (multivariable HR:.77, [95%CI: 0.63–0.96], *P* = .021) and remained similar for genitourinary cancer (multivariable HR:.81, [95%CI: 0.64–1.04], *P* = .094). Similar results were noted when we excluded patients who died but the date of death was not available (Supplementary Figure 2). Our findings remained broadly unchanged in the propensity score-matched cohorts (full cohort multivariable HR:.92, [95%CI: 0.84–1.00], *P* = .052; gastrointestinal cancer multivariable HR:.75, [95%CI:0.60–0.94], *P* = .014; genitourinary cancer multivariable HR:.75, [95%CI: 0.57–0.99], *P* = .042, Supplementary Figure 3).

We compared the rates of irAE between groups. Overall, 40.4% (2387/5910) had an irAE with colitis being the commonest (18,4%, 1085/5910). In comparison, we found no difference in the occurrence of potential irAEs between those on RAAS inhibitors and those not on RAAS inhibitors (41% [1388/3426] versus 40% [999/2484], *P* = .8).

### 3.3. Association between RAAS inhibitor type and dose with overall survival

**Fig. 3.**
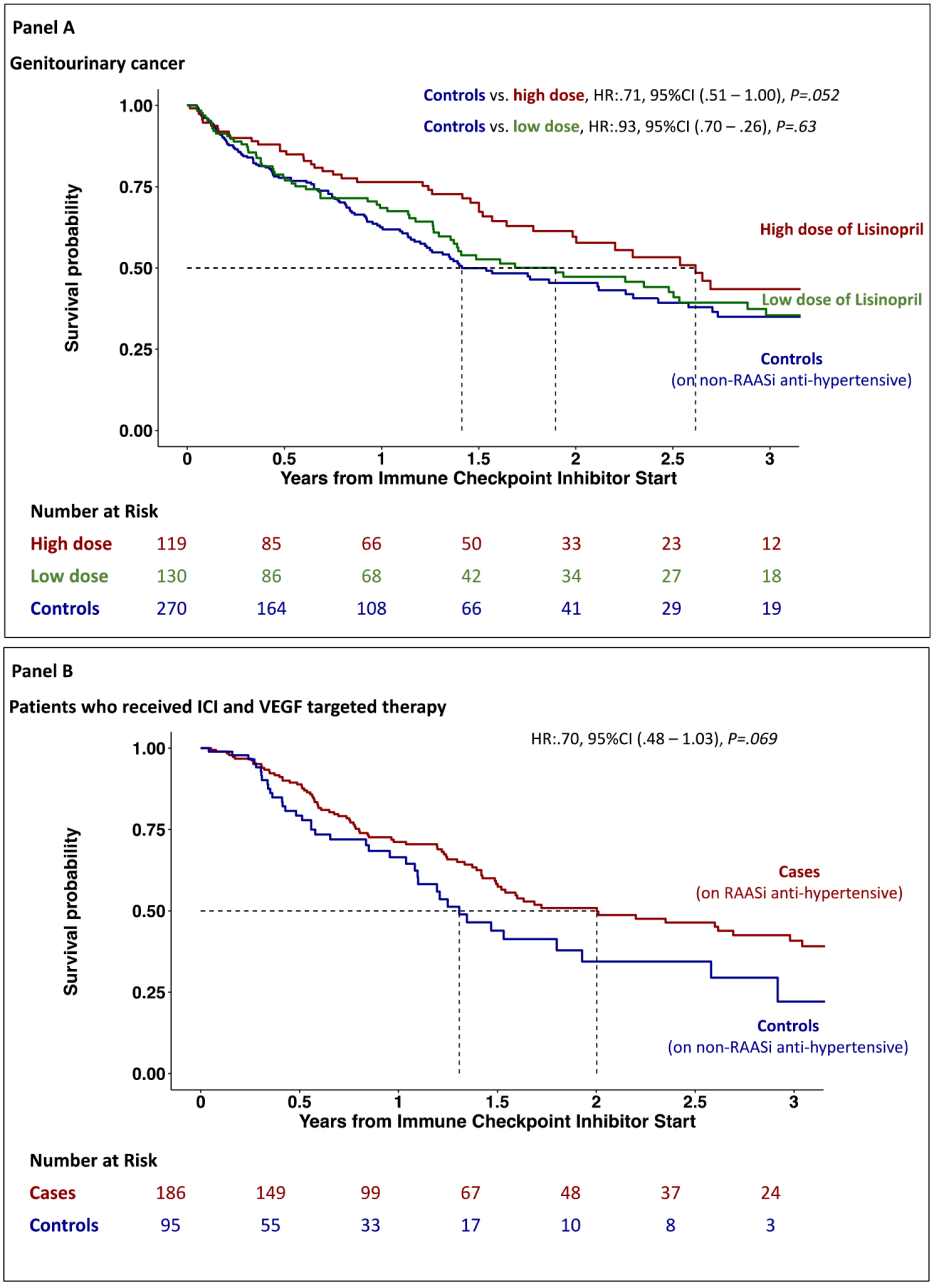
Kaplan–Meier curves of the survival probability over time after starting immune checkpoint inhibitor therapy. Panel A shows patients with genitourinary cancer who were taking Lisinopril and controls patients who were on non–renin–angiotensin–aldosterone system (RAAS) inhibitor anti-hypertensive therapy. Panel B shows overall survival in patients who received ICI and VEGF-targeted therapy.

There was no difference in overall survival among those on an ACE inhibitor versus those on an ARB (univariate HR:1.03, [95%CI: 0.93–1.14], *P* = .59). There was no difference in survival in the full cohort or in patients with gastrointestinal cancer among those taking a high or low dose of lisinopril. However, among patients with genitourinary cancer who were taking a higher dose of lisinopril, there was a trend for improved survival (univariate HR:.71, [95%CI: 0.51–1.00], *P* = .052, Fig. 3/A). Due to the lower sample size for losartan, this analysis was not performed.

### 3.4. Association between RAAS inhibitors and overall survival in patients who also received VEGF therapy

Among the 5910 patients, 863 received VEGF inhibitor therapy anytime, and in 281 patients, there was an overlap between ICI and VEGF inhibitor therapies. Those who received VEGF inhibitor therapy not in overlap with an ICI were excluded from this analysis. Among the 281 patients, 186 were also on a RAAS inhibitor therapy at ICI start. Those who were taking a RAAS inhibitor combined with a VGEF inhibitor had a trend for a better outcome as compared with those who were not (univariate HR:.70, [95%CI: 0.48–1.03], *P* = .069, Fig. 3/B).

## 4. Discussion

Despite having a higher overall risk profile for death, our results suggest that the use of RAAS inhibitor is associated with better overall survival in patients with cancer with hypertension treated with an ICI. Of the common cancer types, patients with gastrointestinal and genitourinary cancer may benefit the most from RAAS inhibitors when treated with an ICI, and patients with these types of cancer appear to have nearly all the benefit. There was no benefit in those with melanoma or thoracic malignancies. There were also no differences in the rates of immune-mediated adverse events between groups and no difference in survival between those on an ACE as compared with an ARB. Furthermore, there was no association between the dose of the RAAS inhibitor and the effect on survival except among patients with genitourinary cancer, where those prescribed a high dose of lisinopril had better outcomes. Finally, our results regarding VEGF inhibitor therapy suggest potential synergy between VEGF-targeted therapy and RAAS inhibitors.

Preclinical data have shown that the RAAS system, via bioactive peptides, have a key role in the tumour microenvironment. In preclinical experiments, inhibiting the RAAS system suppressed cancer cell proliferation, tumour growth, metastasis, and angiogenesis [5,17]. Specifically, the RAAS system can influence the expression of multiple cytokines and growth factors [17], and blockade of the RAAS system can result in decreased expression of VEGF factor, leading to a decrease in VEGF-mediated angiogenesis, a decrease in microvessel density and a decrease in vascular permeability [18,19]. Inhibition of these steps can lead to a normalization of the tumour vasculature and microenvironment [7,17]. The RAAS system can also regulate the tumour microenvironment via transforming growth factor–β, (TGF-β), cancer-associated fibroblasts [5] and tumour-associated macrophages [8]. Inhibition of the RAAS system may block activities mediated by these factors and reduce solid stress, resulting in a less immunosuppressive microenvironment [4,5]. The use of RAAS inhibitors in cancer patients in conjunction with chemo-radiotherapy has been associated with better outcomes in several cancer types (e.g. pancreatic, ovarian, kidney, colorectal, liver, lung and brain) [5,9,10,20–24]. However, some studies found no benefit, therefore suggesting a cancer-type-specific effect. In our study, when analysing patients who received VEGF targeted therapy and ICI in overlap, we found that those who were on a baseline RAAS inhibitor had better outcomes – consistent with retrospective studies in renal carcinoma patients [25–27]. Therefore, there may be an additional synergy between RAAS inhibitors and VEGF targeted therapy in patients receiving ICI therapy.

During the last decade, ICIs have revolutionized cancer treatment, and data suggest that about 50% of cancer patients within the United States may be eligible for treatment with an ICI [3]. However, not all cancer patients respond to this type of therapy [28], and multiple approaches are being tested to improve the response rate [29]. Data regarding the concurrent use of RAAS and ICI are limited. Retrospective studies with small sample sizes have suggested that the concurrent use of RAAS inhibitors may benefit ICI patients with metastatic urothelial carcinoma and non–small-cell lung cancer [15,16]. Jain et al. evaluated patients with metastatic urothelial carcinoma from two centres within the United States (*N* = 178 and *N* = 101), and the effects of the concomitant use of RAAS inhibitors and ICI therapy were analysed. They found that, in univariate analysis, patients who were treated with a RAAS inhibitor had improved tumour regression rates (odds ratio = 3.32; 95% CI, 1.22–9.06; *P* = .019) and a tendency for better overall survival (HR = 0.37; 95% CI, 0.12–1.15; *P* = .051). Our findings are complementary and significantly additive. In our study, when analysed by cancer type, we found that the use of RAAS inhibitors was associated with better overall survival in gastrointestinal and genitourinary cancers treated with ICIs. In another retrospective study, Tozuka et al. among 256 patients with lung cancer, reported improved progression free survival (HR = 0.59, 95%CI, 0.40–0.88) and a trend for lower mortality (HR = 0.71, 95%CI, 0.45–1.11) in those on a RAAS inhibitor [15]. We found no difference in overall survival among patients with thoracic malignancies who were on a RAAS inhibitor as compared with those who were treated with other types of anti-hypertensive medications. RAAS inhibitors in combination with ICI therapy may improve progression free survival in some patients as shown by Tozuka et al., but overall survival was not improved in that study or in our study.

RAAS inhibitors are low-cost, safe and widely prescribed anti-hypertensive drugs [30]. These are widely used and the side effect profile is well known. Up to 70% of patients treated with ICI therapy may experience an irAEs [31]. In our cohort, the occurrence of irAEs was not higher in patients who were concomitantly taking RAAS inhibitors. Given its potential implications for improving overall survival or maybe response rates, further prospective studies in several cancer types are warranted. Based on our results, RAAS inhibitor therapy may have more significant impact on outcomes among patients with gastrointestinal or genitourinary cancers. Indeed, a randomized clinical trial is currently testing potential benefit of adding losartan to chemo-radiation and anti-PD1 antibody in locally advanced pancreatic cancer (NCT03563248) [11]. Our results are similar to a recently published paper, where patients with metastatic urothelial carcinoma were analysed, and the concomitant use of RAAS inhibitors and ICI therapy was associated with a higher rate of tumour regression [16].

### 4.1. Limitations

This was a retrospective hospital network study, which was not designed to provide biological and mechanistic insights. However, our cohort of ICI-treated patients is over 20 times larger than any previously published cohorts, with the inclusion of several cancer types. Patients who were concomitantly taking a RAAS inhibitor at ICI start were older and had more comorbidities. We have performed multivariable analysis and propensity score matching to account for the potential confounding caused by the different risk profiles among the groups. We believe that since patients who were on a RAAS inhibitor had more comorbidities, and that if there is bias in our study, then it is against our findings. Moreover, we have also performed two additional sensitivity analyses where a 1:1 propensity score matching was performed, and in another sensitivity analysis, patients who died but no date of death was available were excluded. In these analyses, our results remained similar.

## 5. Conclusion

In this large, retrospective cohort study, patients with hypertension who were concomitantly taking a low-cost RAAS inhibitor during ICI therapy not only had better overall survival, but also did not develop more irAEs. The benefit of RAAS inhibitors was most pronounced among patients with gastrointestinal and genitourinary cancers. Our results further suggest a dose-dependent effect of RAAS inhibitors. These drugs may also potentiate the effect of the combination of ICI and VEGF-targeted therapy. Prospective randomized trials are warranted to further evaluate and specify the benefit of RAAS inhibitors in patients with cancer who receive ICI therapy.

## Supplementary Material

Supplemental Material

## Figures and Tables

**Table 1:** Baseline characteristics of cases and controls. Cases: patients treated with a renin–angiotensin–aldosterone system inhibitors for hypertension. Controls: patients treated with non–renin–angiotensin–aldosterone system inhibitors for hypertension.

			Cases (N = 3426)	Controls (N = 2484)	P Value
Demographic					
Sex – no. (%)					.001
Male	2093	(61)	1389	(56)	
Female	1333	(39)	1095	(44)	
Age – yrs mean. (SD)	69	(10)	66	(12)	.001
Race or ethnic group – no. (%)					
White	3108	(93)	2256	(92)	.7
Asian	80	(2.4)	70	(2.9)	
Black or African-American	98	(2.9)	67	(2.7)	
Other	69	(2.1)	50	(2.0)	
Clinical variables – mean. (SD)					
Body mass index – kg/m^2^	27.2	(6.1)	26.74	(5.7)	<.001
Systolic blood pressure - mmHg	132.2	(19.1)	128.1	(18.5)	<.001
Cancer types – no. (%)					
Breast	88	(2.7)	98	(4.1)	<.001
Gastrointestinal	381	(11)	275	(11)	
Genitourinary	491	(15)	270	(11)	
Gynaecological	163	(4.9)	77	(3.2)	
Head and neck	288	(8.7)	206	(8.6)	
Haematological	113	(3.4)	104	(4.3)	
Melanoma	555	(17)	358	(15)	
Neurological	111	(3.3)	129	(5.4)	
Sarcoma	33	(10)	24	(10)	
Thoracic	1092	(33)	858	(36)	
Cardiovascular risk factors – no (%)					
Diabetes mellitus	954	(28)	308	(12)	<.001
Smoking current or prior	1325	(39)	1016	(41)	.082
Hyperlipidaemia	2143	(63)	1161	(47)	<.001
Renal disease	690	(20)	311	(13)	<.001
Cardiovascular medications – no. (%)					
Beta-blockers	1915	(56)	1829	(74)	<.001
Calcium channel blockers	1385	(40)	882	(36)	<.001
Statins	2116	(62)	1011	(41)	<.001
Aspirin	1888	(55)	972	(39)	<.001
Prior cancer therapy – no. (%)					
Anthracyclines	178	(5.2)	117	(4.7)	.4
5 fluorouracil	361	(11)	223	(9.0)	.047
Platin-based therapy	1385	(40)	1054	(42)	.12
Number of immune checkpoint inhibitor cycles – no, (IQR)	5	(2–11)	5	(2–10)	.2
Immune checkpoint inhibitor type – no. (%)					
Monotherapy					<.001
Programmed death-ligand-1	483	(14)	285	(11)	
Cytotoxic-T-lymphocyte-associated protein 4	144	(4.2)	72	(2.9)	
Programmed death-protein 1	2554	(75)	1929	(78)	
Combination therapy					
Cytotoxic-T-lymphocyte-associated protein 4/Programmed death protein 1	245	(7.2)	198	(8.0)	
RAAS inhibitor type – no. (%)					
Benazepril	77	(2.3)			
Candesartan	16	(0.5)			
Captopril	19	(0.6)			
Enalapril	70	(2.0)			
Fosinopril	5	(0.2)			
Irbesartan	73	(2.1)			
Lisinopril	2077	(60.6)			
Losartan	748	(21.8)			
Moexipril	6	(0.2)			
Olmesartan	56	(1.6)			
Perindopril	5	(0.2)			
Quinapril	38	(11)			
Ramipril	29	(0.9)			
Telmisartan	11	(0.3)			
Trandolapril	3	(0.1)			
Valsartan	193	(5.6)			

**Table 2:** Baseline laboratory variables of cases and controls. Cases: patients treated with a renin–angiotensin–aldosterone system inhibitors for hypertension. Controls: patients treated with non–renin–angiotensin–aldosterone system inhibitors for hypertension.

	Cases (N = 3426)			Controls (N = 2484)			P Value
	Data available	Mean	SD	Data available	Mean	SD	
Haemoglobin (g/dL)	2659	11.87	1.97	1917	11.92	1.91	<.001
White blood count (thousand/uL)	2567	7.94	9.00	1916	7.81	5.32	<.001
Creatinine	3113	1.03	0.58	2231	0.94	0.41	<.001
Total cholesterol (mg/dL)	927	162.3	42.8	511	170.8	47.0	<.001
Low-density lipoprotein (mg/dL)	854	85.48	33.6	462	94.59	35.64	<.001
High-density lipoprotein (mg/dL)	856	49.35	17.33	459	50.75	18.54	<.001

## Data Availability

The data sets during and/or analysed during the current study available from the corresponding author on reasonable request.
