# Supplementary material for: Renin–angiotensin–aldosterone system inhibitors and survival in patients with hypertension treated with immune checkpoint inhibitors
Source: Eur J Cancer. Author manuscript; Available in PMC 2022 Oct 30. (PMC9618285; doi:10.1016/j.ejca.2021.12.024)

SUPPLEMENTAL MATERIAL

This appendix has been provided by the authors to give readers additional information about their work

Supplement to **Renin-Angiotensin-Aldosterone System Inhibitors and Survival in Patients Treated with Immune Checkpoint Inhibitors**

Zsofia D. Drobni, MD[1,2], Olivier Michielin MD, PhD[3,4], Thiago Quinaglia MD[2], Daniel A. Zlotoff, MD PhD[5], Leyre Zubiri, MD PhD[6], Hannah K Gilman, BS[2], Sama Supraja, BS[2], Bela Merkely, MD[1], Veronika Muller, MD[7], Ryan J. Sullivan, MD[6], Kerry L. Reynolds, MD[6], Michael J. Pittet, PhD[8], Rakesh K. Jain, PhD[9], Tomas G. Neilan, MD, MPH[2,5]

1, Heart and Vascular Center, Semmelweis University, Budapest, Hungary
2, Cardiovascular Imaging Research Center (CIRC), Department of Radiology and Division of Cardiology, Massachusetts General Hospital, Harvard Medical School, Boston, MA, USA
3, Oncology Department, Precision Oncology Center, Lausanne, Switzerland
4, Oncology Department, Lausanne University Hospital, Lausanne, Switzerland
5, Cardio-Oncology Program, Division of Cardiology, Department of Medicine, Massachusetts General Hospital, Harvard Medical School, Boston, Massachusetts
6, Division of Oncology and Hematology, Department of Medicine, Massachusetts General Hospital, Harvard Medical School, Boston, MA, USA
7, Department of Pulmonology, Semmelweis University, Budapest, Hungary
8, Department of Pathology and Immunology, University of Geneva, Geneva, Switzerland
9, Edwin L. Steele Laboratories, Department of Radiation Oncology, Massachusetts General Hospital and Harvard Medical School, Boston, MA, USA

Table of Contents

Supplementary Table 1. Baseline characteristics of cases and controls. Cases: patients treated with a renin-angiotensin aldosterone system inhibitors for hypertension. Controls: patients treated with non- renin-angiotensin aldosterone system inhibitors for hypertension. Patients without available date of death were excluded. *3*

Supplementary Figure 1. Kaplan Meier curves of the survival probability in each cancer type in the full cohort. *5*

Supplementary Figure 2. Kaplan Meier curves of the survival probability in all types of cancer, genitourinary and gastrointestinal, excluding those who died but no date of death available. *6*

Supplementary Figure 3. Kaplan Meier curves of the survival probability in Kaplan Meier curves of the survival probability in all types of cancer, genitourinary and gastrointestinal in the propensity score matched cohort. *7*

Supplementary Table 1. Baseline characteristics of cases and controls. Cases: patients treated with a renin-angiotensin aldosterone system inhibitors for hypertension. Controls: patients treated with non- renin-angiotensin aldosterone system inhibitors for hypertension. Patients without available date of death were excluded.

	Cases (N = 3,426)		Controls (N = 2,484)		*P* Value
**Demographic**					
Sex – no. (%)					
Male	1,897	(61)	1,244	(55)	< 0.001
Female	1,207	(39)	1,003	(46)	
Age – yrs mean. (SD)	68	(10)	66	(12)	< 0.001
Race or ethnic group – no. (%)					
White	2,823	(93)	2,046	(93)	0.9
Asian	77	(2.5)	60	(2.7)	
Black or African American	85	(2.8)	58	(2.6)	
Other	57	(1.9)	47	(2.1)	
**Clinical variables – mean. (SD)**					
Body mass index - kg/m^2	28.13	(6.11)	26.78	(5.74)	< 0.001
Systolic blood pressure - mmHg	132.3	(19.05)	126.5	(17.91)	< 0.001
**Cancer types – no. (%)**					< 0.001
Breast	78	(2.6)	90	(4.1)	
Gastrointestinal	345	(12)	243	(11)	
Genitourinary	449	(15)	247	(11)	
Gynecological	150	(5.0)	68	(3.1)	
Head and Neck	262	(8.7)	189	(8.7)	
Hematological	100	(3.3)	91	(4.2)	
Melanoma	508	(17)	333	(15)	
Neurological	95	(3.2)	114	(5.3)	
Sarcoma	25	(0.8)	21	(1.0)	
Thoracic	1,987	(33)	773	(36)	
**Cardiovascular risk factors – no (%)**					
Diabetes mellitus	852	(27)	284	(13)	< 0.001
Smoking current or prior	1,183	(38)	913	(41)	0.062
Hyperlipidemia	1,923	(62)	1,047	(47)	< 0.001
Renal disease	604	(19)	279	(12)	< 0.001
**Cardiovascular medications – no. (%)**					
Beta-blockers	1,729	(56)	1,651	(73)	< 0.001
Calcium channel blockers	1,237	(40)	785	(35)	< 0.001
Statins	1,900	(61)	917	(41)	< 0.001
Aspirin	1,685	(54)	875	(39)	< 0.001
**Prior cancer therapy – no. (%)**					
Anthracyclines	155	(5.0)	107	(4.8)	0.7
5 fluorouracil	326	(11)	199	(8.9)	0.046
Platin based therapy	1,264	(41)	939	(42)	0.4

**Immune checkpoint inhibitor type – no. (%)**					< 0.001
Monotherapy					
Programmed death-ligand-1	446	(14)	261	(12)	
Cytotoxic-T-Lymphocyte associated protein 4	130	(4.2)	63	(2.8)	
Programmed death-protein 1	2,299	(74)	1,740	(77)	
Combination therapy					
Cytotoxic-T-Lymphocyte associated protein 4/Programmed death protein 1	229	(7.4)	183	(8.1)	
Number of cycles of ICI – no, (IQR)	5	(2 – 11)	5	(2 – 10)	0.2

Supplementary Figure 1. Kaplan Meier curves of the survival probability in each cancer type in the full cohort. (Results for any type of cancer, gastrointestinal and genitourinary are reported in the main manuscript)

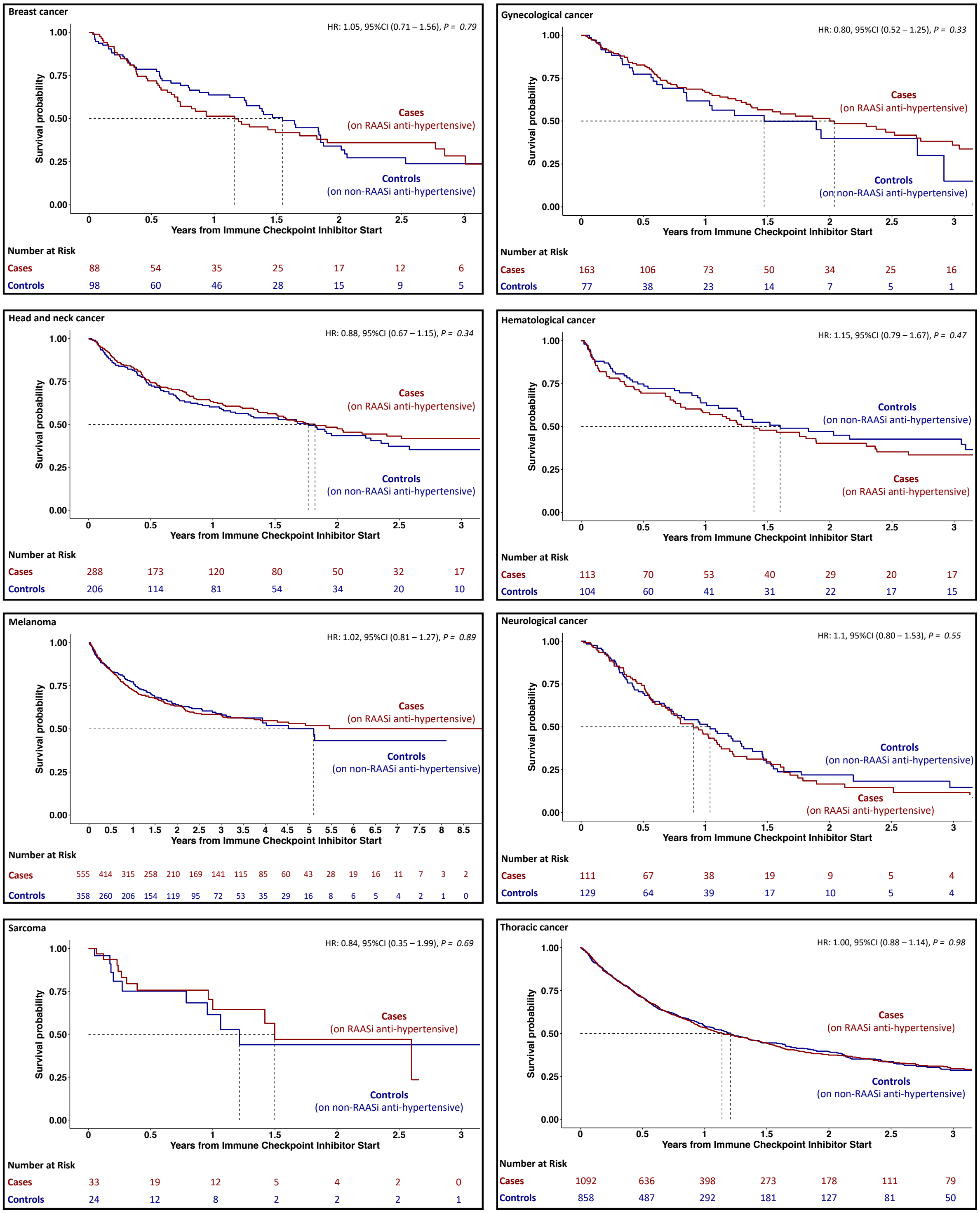

Supplementary Figure 2. Kaplan Meier curves of the survival probability in all types of cancer, genitourinary and gastrointestinal, excluding those who died but no date of death available. *: Adjusted hazard ratios for age, gender, body mass index, congestive heart failure, diabetes, renal disease, liver disease and smoking.

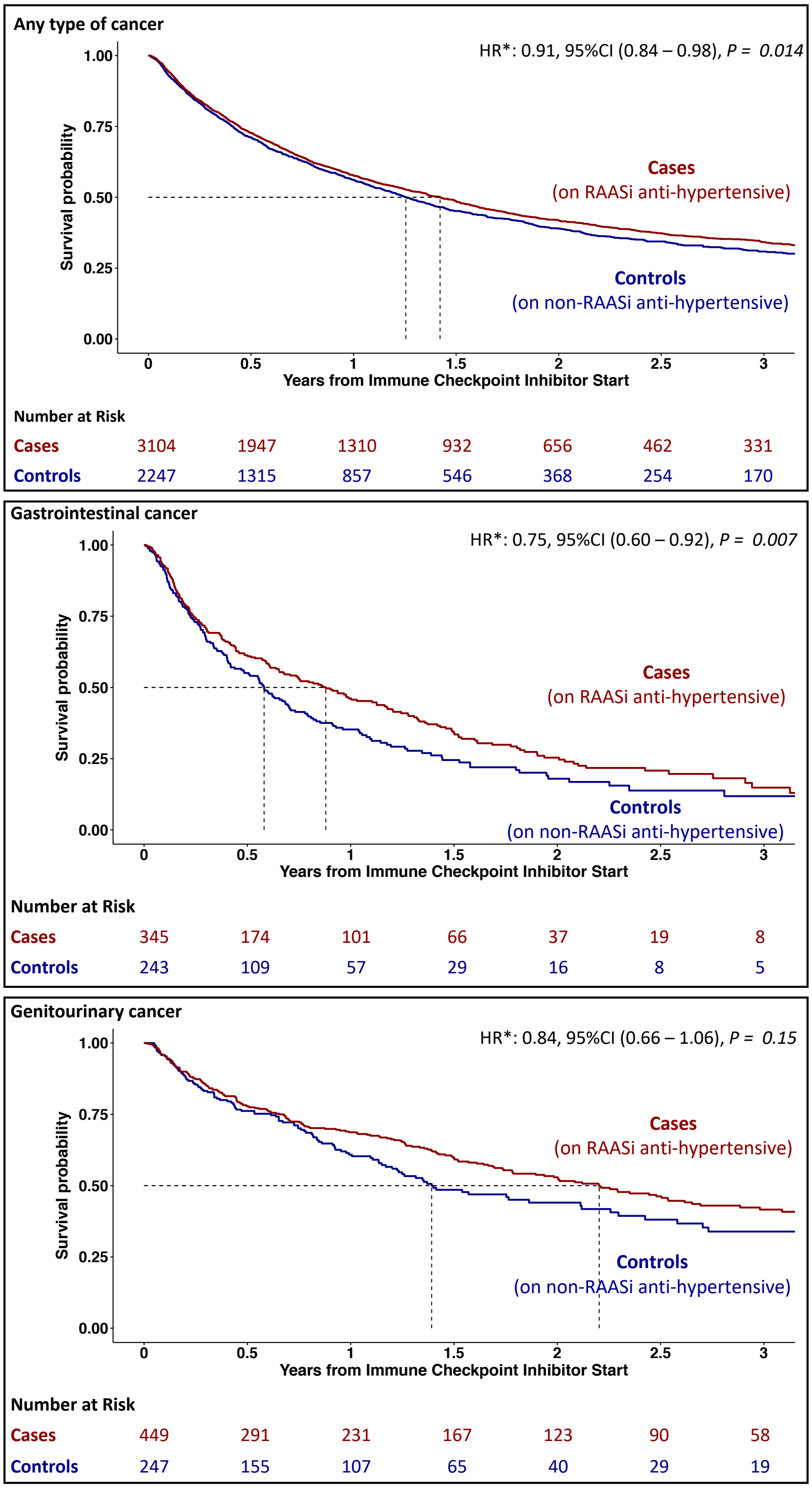

Supplementary Figure 3. Kaplan Meier curves of the survival probability in Kaplan Meier curves of the survival probability in all types of cancer, genitourinary and gastrointestinal in the propensity score matched cohort. *: Adjusted hazard ratios for age, gender, body mass index, congestive heart failure, diabetes, renal disease, liver disease and smoking.

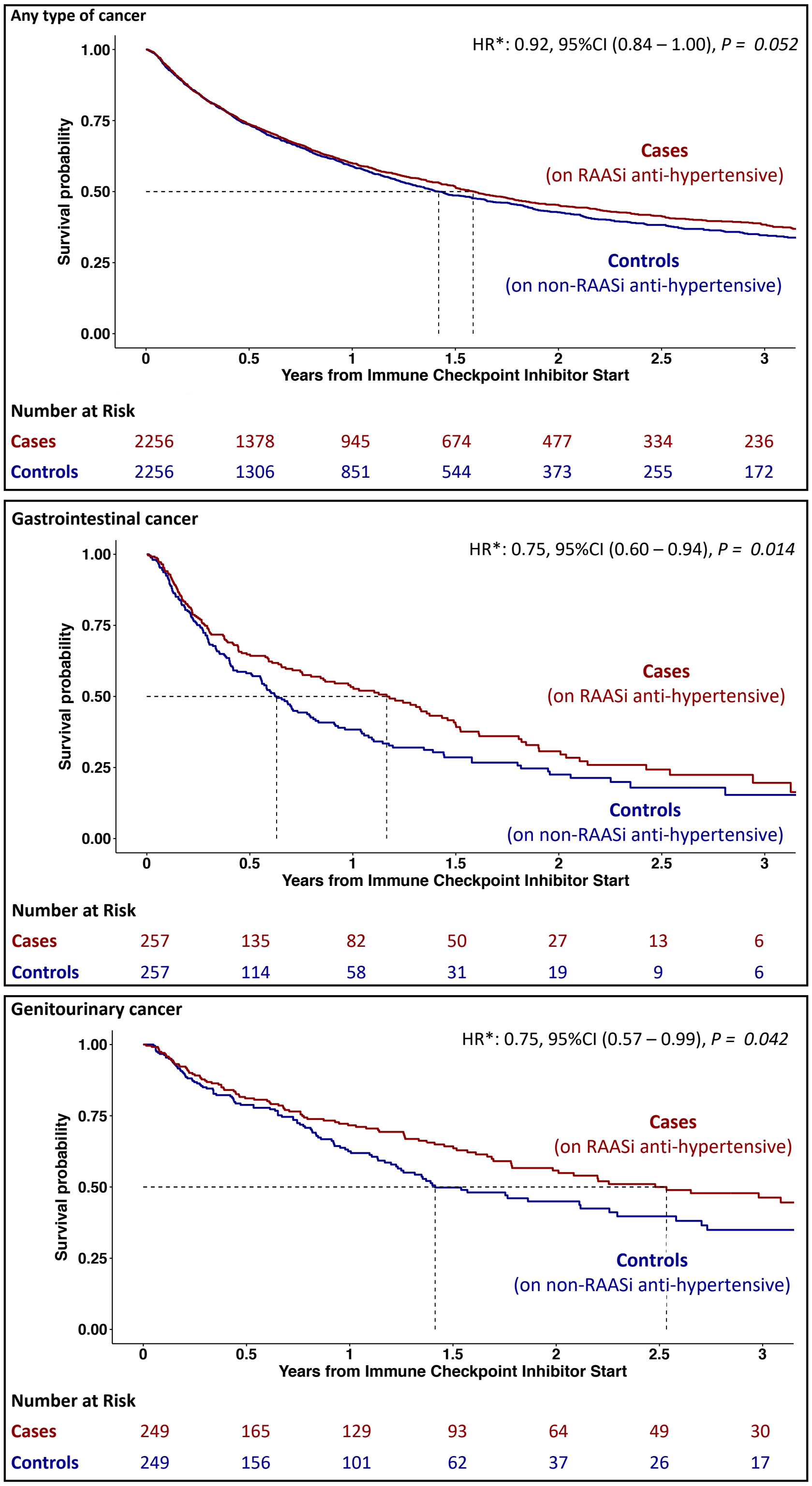

Supplement: Supplemental Material [file NIHMS1843272-supplement-Supplemental_Material.pdf]
